# Exercise-induced blood flow in relation to muscle relaxation period

**DOI:** 10.1186/1476-5918-6-5

**Published:** 2007-05-09

**Authors:** Fumiko Ohmori, Shizuyo Shimizu, Atsuko Kagaya

**Affiliations:** 1 National Institute of Fitness and Sports in Kanoya, 1 Shiromizu, Kanoya, Kagoshima 891-2393, Japan; 2 Institute of Physical Education, Keio University, 4-1-1 Hiyoshi, Kohoku, Yokohama, Kanagawa 223-8521, Japan; 3 Research Institute of Physical Fitness, Japan Women's College of Physical Education, 8-19-1 Kitakarasuyama, Setagaya, Tokyo 157-8565, Japan

## Abstract

**Background:**

Dynamic exercise is characterized by relaxation periods between contractions. The relaxation period should be considered as a causal factor for determining the magnitude of blood flow during dynamic exercise. The purpose of this study was to investigate the effect of muscle relaxation periods determined by the response of each subject on the exercise-induced blood flow response.

**Methods:**

Seven healthy female subjects performed dynamic plantar flexions twice in succession; the duration of each flexion was 1- s and they were performed at an intensity of 15%, 30% and 50% of the maximal voluntary contraction (MVC). Based on the blood flow response after a single contraction, we set up intervals between two successive contractions; the intervals corresponded to 50% (pre-T_peak_), 100% (T_peak_), and 150% (post-T_peak_) of the time required to reach peak blood flow.

**Results:**

In all the conditions, upon cessation of the contraction, there was a progressive, beat-by-beat increase in the blood flow through the popliteal artery that peaked by the 5^th ^cardiac cycle. Peak values of blood flow achieved after exercise were significantly higher at pre-T_peak _than at T_peak _and post-T_peak _(p < 0.05).

**Conclusion:**

The result indicate that at three intervals based on the time taken to reach the peak value, the highest blood flow value was obtained at the pre-T_peak _interval.

## Background

The blood flow to an active muscle changes depending on the exercise intensity, contraction frequency, contraction-to-relaxation duty cycle, etc [1-9]. The blood flow increases markedly during the relaxation phase of dynamic exercise, whereas it remains at a lower level during the contraction period [10-14]. Thus, the magnitude of the blood flow during the relaxation phase of the dynamic exercise determines the magnitude of blood supply to the exercising muscles. Further, the manner in which an exercise protocols increases the blood flow during the relaxation phase of dynamic exercise needs to be clarified.

An earlier study on contraction-to-relaxation duty cycle indicated that the blood flow to an active muscle during dynamic exercise reflects the influence of alteration in the duration of the relaxation phase, rather than the effect of altering the contraction rate [9]. It was also reported that a second contraction of the same intensity during the period of increased blood flow due to the first contraction induced a greater increase in the blood flow than that caused by the first contraction alone [15]. Therefore, the relaxation time between successive contractions should be a causal factor for the determination of blood flow during dynamic exercise. Hence, the time courses of changes in the blood flow immediately after dynamic contraction should be clarified.

The immediate post-exercise flow was approximately analogous to an interpolated blood flow during relaxation [10,13]. Moreover the blood flow early in recovery after exercise differed between subjects, including athletes and non-athletes [16]. Based on these findings, it is reasonable to hypothesize that exercise-induced hyperemia may vary between the subjects depending on the time taken to reach peak blood flow. To elucidate the changes in blood flow induced by the contraction-to-relaxation duty cycle, the relationship between the changes in the blood flow during relaxation phase and augmentation of blood flow during exercise should be clarified. However, the effect of relaxation time on the time course of changes in the blood flow has never been assessed previously.

Therefore, the aim of this study was to elucidate the effect of muscle relaxation periods that were determined from the blood flow response of each subject on the exercise-induced blood flow. The following approaches were employed. The three intervals were chosen based on the time taken to reach peak value of blood flow after a single contraction of plantar flexion exercise. Alternations in the blood flow after contractions were studied by comparing the 3 different time durations between contractions–the time corresponding to peak value of blood flow, before reaching the peak value, and after the peak value of blood flow was reached.

## Methods

### Subjects

Seven physically active women participated in the study after giving their informed consent. The age, body height and body mass (means ± SD) of the subjects were 21.9 ± 0.7 years, 162.5 ± 5.8 cm and 55.4 ± 5.3 kg, respectively. All the subjects were free of medical problems.

### Experimental protocol

#### Exercise protocol

All the experiments were performed in the supine position. To fix the entire distance between shoulder and foot, the subjects were stabilized in a fixed position by a using a padded support plate. Each subject placed their respective right foot on the pedal of the ergometer with ankle and knee joints angle at 90° and 180°, respectively. The subject pressed the pedal with the ball of the foot to extend the ankle joint to 100°, and then the subject relaxed their foot to return the ankle joint to 90°. Dynamic plantar flexion was performed against the loads adjusted to 15%, 30%, and 50% of the maximal voluntary contraction (MVC). The MVC of the plantar flexors was determined isometrically and the average of 3 of 5 trials, i.e., excluding the highest and lowest values, was used as the representative MVC to standardize the load. The duty cycle comprised 0.5-second lifting, 0.5-second hold, and 0.5-second unloading. The cadence was determined using an auditory metronome. Prior to the experiments, the experimental approach was explained to all the subjects; the subjects practiced the exercise to ensure that they could maintain the contraction-relaxation schedule and cadence. The experiment was conducted in a room with the temperature and relative humidity set at 24°C and 60%, respectively.

### Determination of the relaxation time

**Figure 1:**
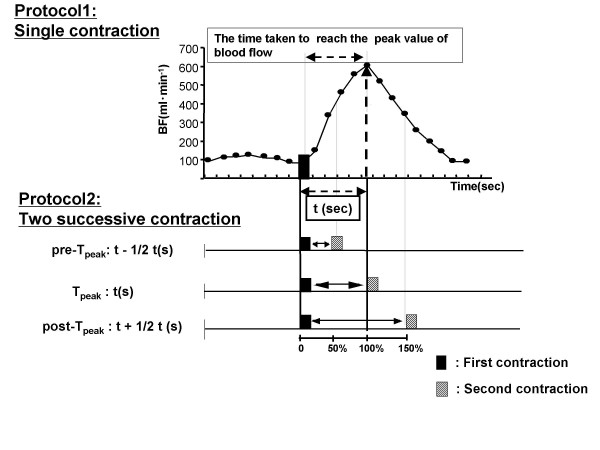
**Exercise protocol**. We set up the time intervals based on the time taken to reach the peak value of blood flow. In the first experiment, we investigated popliteal artery blood flow after a single 1-second contraction of plantar flexion exercise. Immediately after the cessation of contraction, the blood flow began to increase markedly and reached its peak value. The intervals between the contractions used in this study were 50%, 100%, and 150% of the time taken to reach the peak value.

Two experimental protocols were tested in this study (Figure 1). In the first experiment, we investigated the popliteal artery blood flow after a single contraction during plantar flexion exercise to determine the time at which peak blood flow was achieved, i.e., the time taken to reach the peak value of blood flow after contraction.

In the second experiment, two successive plantar flexor contractions were performed at three intervals that were based on the results of the first experiment. The intervals between contractions used in this study corresponded with the following time durations: to 1) The time taken to reach the peak blood flow (T_peak_), 2) the interval preceding T_peak _(pre-T_peak_), and 3) the interval following T_peak _(post-T_peak_). The time corresponding to pre-T_peak _and post-T_peak _were calculated as follows: pre-T_peak _= t – 1/2t, post-T_peak _= t + 1/2t, where t was the time taken to reach the peak value of blood flow.

### Physiological measurement

The mean blood velocity and the vessel diameter of the popliteal artery were measured by using a Doppler and B-mode ultrasound method (HP SONOS 1000, USA). A 7.7 MHz linear array transducer was placed approximately 1 cm above the bifurcation of the popliteal artery into the anterior and posterior tibial arteries. The sampling volume was maintained at 8.9 mm and the angle of the beam to determine the direction of flow of blood was adjusted automatically to 60°.

The diameter of blood vessel based on the relative time periods of the systolic (1/3) and the diastolic (2/3) phases of the cardiac cycle was assumed to be the most representative of the diameter size for each cardiac cycle, and it was utilized for determining the cross-sectional area of the vessel (A = πr^2^, where r is the radius of the vessel); this was used to calculate the blood flow.

Beat-by-beat popliteal artery blood flow was calculated by multiplying FI, HR, and πr^2^, where FI is the flow integral during each cardiac cycle and HR is the heart rate obtained from the R-R interval of the electrocardiogram (ECG).

### Statistical analysis

The group values are expressed as mean ± SE. Differences among means obtained from the 3 intervals were evaluated by using a one-way ANOVA with post hoc comparison using Fischer's PLSD. Difference between the blood flow during the relaxation period and post-contraction were analyzed using t test for paired samples. A p value of less than 0.05 was considered statistically significant.

## Results

### Blood flow and time to reach peak blood flow after a single contraction

**Figure 2:**
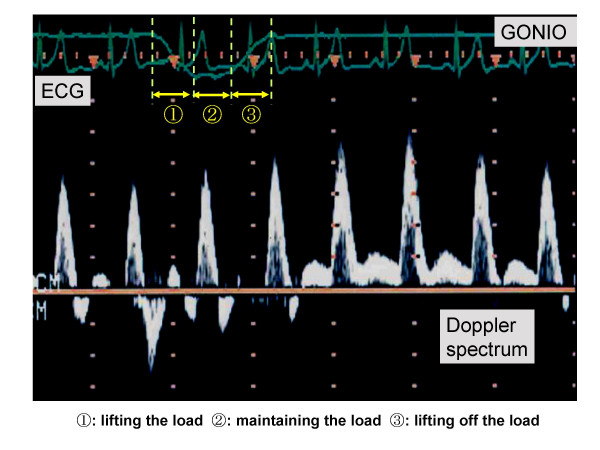
Blood velocity signal before, during and after a single contraction.

Following the initiation of a decrease in tension after the 0.5-s hold, the blood flow began to increase immediately. Moreover, it continued to increase even after the developed tension returned to baseline and reached the peak values in 3.8 – 4.7 seconds (mean: 4.0 ± 0.2) (Figure 2). Table 1 shows the time taken to reach the peak blood flow and the coefficients of variation of the time to reach the peak blood flow for each subject at 3 intensities. There were no significant differences in the time taken to reach the peak value of blood flow among the 3 intensities; however, there were significant differences in the peak blood flow achieved, at different exercise intensities, and those obtained during exercise at 30% and 50% MVC were significantly higher than that obtained at 15% MVC (p < 0.05: Table 1).

**Table 1:** Peak blood flow and the time taken to re ach the peak blood flow after a single contraction

	15%MVC	30%MVC	50%MVC
Peak BF (ml/min Mean ± S	**149 ± 15**	**416 ± 41**	**488 ± 55**
The time taken to reach the peak BF (s)	**3.8 ± 0.3**	**4.7 ± 0.2**	**3.9 ± 0.2**
The coefficients of variation (%)	**23**	**12**	**14**

Data are obtained from after single contraction. Peak value of blood flow at 30% and 50% MVC were significantly higher than that at 15% MVC. Peak BF: Peak value of blood flow.

*p < 0.05 as compared to 15% MVC

**Figure 3:**
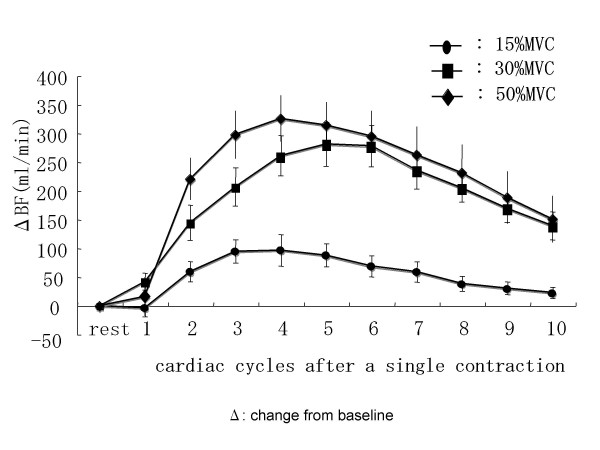
Beat-by-beat blood flow after a single contraction.

The average of the beat-by-beat blood flow after a single contraction in the 7 subjects at all intensities is illustrated in Figure 3. The blood flow reached its peak value by the 4 ^th ^or 5 ^th ^cardiac cycle.

The relaxation time calculated using the time to reach peak blood flow after single contraction is listed in Table 2.

**Table 2:** Determination of the relaxation time

	**pre-T**_**peak **_(sec)	**T**_**peak **_(sec)	**post-T**_**peak **_(sec)
**15% MVC**	**2.0 ± 0.2**	**3.9 ± 0.4**	**5.7 ± 0.4**
**30% MVC**	**2.3 ± 0.1**	**4.6 ± 0.3**	**6.7 ± 0.3**
**50% MVC**	**2.1 ± 0.1**	**3.9 ± 0.2**	**5.8 ± 0.4**
			(Mean ± SE)

Data are the relaxation time calculated using the time to reaching peak blood flow after a single contraction.

### Blood flow and time to reach the peak blood flow after two successive contractions

**Figure 4:**
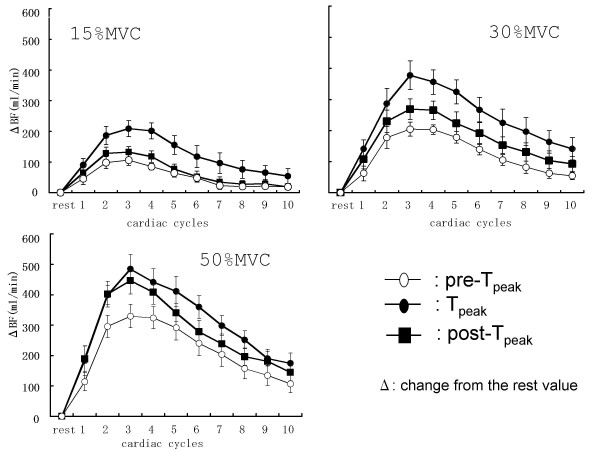
**Beat-by-beat blood flow after two successive contractions**. At all contraction frequencies and intensities, a beat-by-beat progressive increase in the popliteal artery blood flow was observed following the cessation of contraction, and the peak value was obtained during the third cardiac cycle.

The beat-by-beat blood flow after contractions at all contraction frequencies and intensities are illustrated in Figure 4. At all contraction frequencies and intensities, the peak value of blood flow was observed during the third cardiac cycle after two successive.

**Figure 5:**
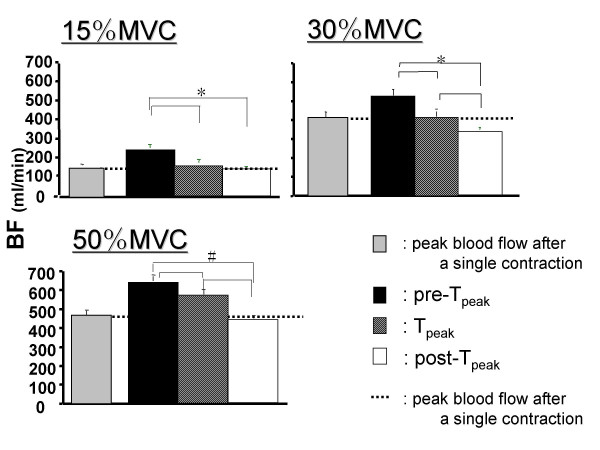
**Peak blood flow in exercise at three different relaxation times**. The pre-Tpeak interval showed the highest blood flow value and the value decreased as the relaxation period increased. *p < 0.05 pre-T_peak _compared with T_peak _and post-T_peak _at 15% MVC and during the three relaxation intervals at 30% MVC. #p < 0.01 during the three relaxation period at 50% MVC.

The peak value of blood flow immediately after two successive contractions are shown in Figure 5. A comparing of the three different intervals reveals that the highest blood flow values were obtained after the second contraction at the pre-T_peak _interval at all intensities (p < 0.05). In contrast, the blood flow value was the lowest during the post-T_peak _interval further, the blood flow after the contraction in the post-T_peak _interval was lowers than that after a single contraction at 15% and 30%MVCs.

## Discussion

The major finding of this study was that the blood flow was augmented to a greater extent when the second contraction occurred prior to the time of the peak hyperemic response. The contraction interval in this study was based on using the time taken to reach peak blood flow after a single contraction. We set up at three intervals using the time duration reaching peak value; the time shorter and longer the time reaching peak value after a single contraction. As a result, the highest blood flow value was obtained from interval using the time before reaching peak value after a single contraction.

As reported by previous studies, the blood flow after a single contraction began to increase markedly immediately after contraction [15,17,18]. After a single contraction, a beat-by-beat increase in the blood flow was observed, and the peak value was obtained between the 4 ^th ^and 5 ^th ^cardiac cycles. At all contraction intervals and intensities, after two successive contractions, the blood flow showed a progressive, beat-by-beat increase peaked by the 3^rd ^cardiac cycle. These results are similar to those reported by Tschakovsky et al [19]. Although all subjects required a similar number of cardiac cycles to reach the peak blood flow, the time taken to reach peak blood flow differed between subjects because of differences in the R-R intervals of the subjects.

In this study, immediately after the tension in the muscle began to decrease during unloading, the blood flow began to increase and continued to do despite the return of the tension to resting levels. This rapid increase is most likely a consequence of the mechanical effects of the muscle pump on alterations of the perfusion pressure gradient across the capillary bed [20]. The rapid vasodilation after a single contraction was detectable within approximately 0.5 – 2 s [15,19]. Considering from the time taken to reach the highest blood flow (3.8– 4.7 s), we cannot exclude the possibility of vasodilation mechanism due to the local release of any vasodilators [15,17].

A novel featuring of this study was that the relaxation times for each subject were set up by using relative time based on the time taken to reaching peak value of blood flow after a single contraction. The advantage of the use of relative time is that the timing of the second contraction was adjusted for change in the blood flow changes of each subject. As a result, it indicates that the blood flow will markedly increase during two successive contraction model used in this study if the second contraction comes 50% of time for reaching peak blood flow. Corcondilas et al [15] reported that amount of blood flow increase was different when second contraction was performed four seconds and ten seconds after a single contraction. It is possible to suppose that the effect of the exercise stimulus (mechanical or metabolic stimulus, central command etc) to the vessel caused by a single contraction may be summated when the second contraction comes before post-contraction blood flow reaches the peak. On the other hand, the summation may not occur when the second contraction comes at or after the time for the peak post-contraction hyperemia. Further physiological mechanism remains to be studied.

The three relaxation times that were used in this study corresponded to 2.1– 2.3 s (pre-T_peak_), 3.9– 4.6 s (T_peak_) and 5.7– 5.6 s (post-T_peak_). Among the three relaxation times, the highest blood flow was obtained at the pre-T_peak _contraction interval. This is in agreement with the results of Byström and Kilbon [7] who demonstrated that the blood flow during exercise with short intervals were higher than that during exercise with long intervals. The contraction-to-relaxation duty cycle in this study corresponded to a frequency of 19–20 contractions per min (cpm), 11–13 cpm, and 8–9 cpm at the pre-T_peak_, T_peak_, and post-T_peak _intervals, respectively. Several studies have indicated that an increase in the blood flow and contraction frequency was paralleled at lower contraction frequency [8,21-23]. With regard to higher contraction frequencies, the results obtained were inconsistent with those of some studied that showed it increased blood flow [21,22] and the others showing decreased blood flow [9]. The contraction frequency used in this study was lower than that used in the previous studies. The effect of contraction intervals shorter than that used in the present study on the blood flow remains to be studied.

With respect to the exercise intensities (15%, 30%, and 50% MVC) used in this study, the peak blood flows after contraction increased with the increase in the exercise intensity. This might be either due to the number of muscle fibers recruited [24] or the increased tension produced due to the increase in the exercise intensity [3]. Moreover, Hamann et al [25] indicated that the blood flow response to a single muscle contraction is not solely determined by the rate at which work is performed; muscle fiber recruitment also contributes independently to the changes in the blood flow.

## Conclusion

Dynamic plantar flexion contractions were performed twice successively at three different intervals, which were determined based on the time taken to reach peak value of blood flow after a single contraction. We demonstrated if the second contraction occurs before the peak value of blood flow is reached subsequent to the previous contraction, the increase in the blood flow is higher as compared to the case if the second contraction occurs after the peak blood flow is reached.
